# Chest Trauma Experience: Incidence, associated factors, and outcomes among patients in Saudi Arabia

**DOI:** 10.12669/pjms.37.2.3842

**Published:** 2021

**Authors:** Waseem M. Hajjar, Sami A. Al-nassar, Omar S Almutair, Abdulrahman H. Alfahadi, Nawaf H. Aldosari, Sultan Ayoub Meo

**Affiliations:** 1 Waseem M. Hajjar, Department of Surgery, College of Medicine, King Saud University, Riyadh, Kingdom of Saudi Arabia; 2 Sami A. Al-nassar, Department of Surgery, College of Medicine, King Saud University, Riyadh, Kingdom of Saudi Arabia; 3 Omar S. Almutair, Department of Surgery, College of Medicine, King Saud University, Riyadh, Kingdom of Saudi Arabia; 4 Abdulrahman H. Alfahadi, Department of Surgery, College of Medicine, King Saud University, Riyadh, Kingdom of Saudi Arabia; 5 Nawaf H. Aldosari, Department of Surgery, College of Medicine, King Saud University, Riyadh, Kingdom of Saudi Arabia; 6 Sultan Ayoub Meo, Department of Physiology, College of Medicine, King Saud University, Riyadh, Kingdom of Saudi Arabia

**Keywords:** Chest trauma, Etiology, Patterns, Management of chest trauma, Tertiary

## Abstract

**Background and Objective:**

Worldwide chest trauma is considered one of the leading causes of morbidity and mortality. There is a lack of sufficient information on the etiology, pattern, and management of these injuries in Saudi Arabia. Therefore, the current study was conducted to determine the spectrum of chest trauma and its associated factors among patients admitted to King Khalid University Hospital, Riyadh, Saudi Arabia.

**Methods:**

A quantitative observational cross-sectional analysis was performed, data obtained from the medical records of the chest trauma patients which were admitted in the thoracic surgery unit, King Khalid University Hospital (KKUH), Riyadh, Saudi Arabia from January 2013 to Jan 2019. The records of all these patients were reviewed and data were collected and analyzed prospectively.

**Results:**

A total of 236 patients (male: 87.3%; mean age: 32.4 years) were included in the analyses. The majority of these chest trauma cases (n=205; 86.9%) were caused by road traffic accidents (RTA). Blunt trauma predominated the cases n=225 (95.3%). Ribs fracture had the highest prevalence among the chest injuries with a number of 150 (63.5%) followed by lung contusion 140 (59.3%). Pneumothorax occurred in 131 (55.5%) and hemothorax occurred in 80 (33.8%) with most common indication for emergency thoracotomy. Extra-thoracic injuries involving the head/brain, limbs, and abdominal organs occurred in 189 (80%). 130 (55%) were intubated and ventilated, and almost half of the patients 115 (48.7%) were required a chest tube insertion.

**Conclusions:**

Chest trauma is a major health issue particularly in young male adults and road traffic accidents are the leading cause of chest trauma in Saudi Arabia. Early recognition of the patterns, etiology and appropriate management of trauma reduce the incidence of chest trauma related injuries.

## INTRODUCTION

Chest trauma is usually a public health problem associated with high morbidity and mortality worldwide. It is reported to be one of the major causes of hospitalization and long-term disabilities, accounting for 25% of all trauma-related deaths and 10% of hospital admissions.^1^-^4^ Chest trauma can occur due to a variety of reasons; however, the majority were related to road traffic accidents (RTA) that account for more than 70% of all thoracic injuries.^5^

In the Kingdom of Saudi Arabia (KSA), RTA are one of the highest world-wide. It is very severe with an 8:6 accident to injuries ratio, compared with 8:1 international ratio.^6^ It is established to have greater extent of deaths among high-income countries and cause 4.7% of all mortalities (24 per 100,000 population). KSA has documented 86,000 mortalities and 611,000 injuries in the last two decades. It is considered the kingdom’s main cause of death for 16-30-year-old males. Saudi Ministry of Health (MOH) hospitals state that RTA victims occupy approximately 20% of all beds, and RTA account for 81% of deaths in the hospitals.^6^-^8^

Thoracic trauma comprises around 10-15% of all traumas world-wide with a variety of injuries ranging from simple chest wall contusion or rib fractures to vital organ injury including lung contusion, hemothorax, pneumothorax, flail chest, Broncho-pleural fistula and tracheobronchial rupture. Vast majority of chest trauma cases can be managed conservatively through chest tube insertion, blood transfusion, and intubation; etc., However, few of these cases, which sustained severe chest injuries may require emergency thoracotomy as a definitive management.^7^,^8^ One of the most common intra-thoracic injuries is the pulmonary contusion that results from blunt chest trauma, some of them needs endotracheal intubation, oxygenation and mechanical ventilation.^5^

Various studies described injury per regions, or parts of body involved, and others as multiple system injured. However, the incidence of chest trauma is significantly increasing in Saudi Arabia due to the increased rate of RTA. However, to the best of our knowledge, there is a lack of local studies or literature on the incidence and outcome of such chest trauma associated with RTA from this region in KSA.^8^ This study was conducted to determine the patterns, etiology, management approaches, and outcome of chest trauma in one of the tertiary care hospitals in Riyadh, Saudi Arabia. The findings provide understanding the incidence of chest trauma and to assist in developing appropriate management approaches and awareness programs to minimize such injuries, in addition to adopt a preventive measures and protocols to reduce the chest trauma deaths.

## METHODS

The present quantitative observational cross-sectional study was conducted in the thoracic surgery unit, King Khalid University Hospital (KKUH), King Saud University Riyadh, Saudi Arabia during January 2013 to January 2019. The data obtained from the medical records of the chest trauma patients which were admitted to the thoracic surgery unit, KKUH. The records of all these patients were reviewed and data were collected and analyzed prospectively. All chest trauma patients were included in this study. Patients less than16 years old, lacked or missing the required information, and patients with isolated brain, abdomen and limbs trauma were excluded from the study. Data were categorized as, median, means, range, standard deviation, and proportions and analyzed according to age, gender, etiologies, patterns of injury, and management approaches. Data were analyzed using SPSS (Version-23) software. Patient confidentiality was assured by assigning each participant with a code number to maintain their privacy. Institutional review board (IRB), College of Medicine, King Saud University approval was obtained prior to commence this study.

## RESULTS

A total of 236 chest trauma patients admitted to our unit, male accounted for 206 cases (87.3%); mean age: 32.4± 14.5 years [range 16 - 85 years]; Saudi: 192 [81.3%]), The records of all these patients were reviewed and data were collected and analyzed. Up on arrival to the hospital, 122 (51.7%) patients had life-threatening condition requiring immediate medical attention (Table-I).

**Table-I:** Demographic characteristics of chest trauma cases seen in our institute, Riyadh, Saudi Arabia between 2013-2018.

*Variables*	*n (%)*
Total number of cases	236
***Gender***	
Males	206 (87.3%)
Females	30 (12.7%)
***Nationality***	
Saudi	192 (81.3%)
Non- Saudi	44 (18.7%)
***Condition upon arrival***	
Emergency / Life threatening	122 (51.7%)
Urgent / Not life threatening	98 (41.5%)
Referred	16 (6.8%)

Blunt injury was the major trauma pattern observed in the cases (n=225, 95.3%). While more than half of the cases 130 (55%) were managed through endo tracheal intubation and Mechanical ventilation, 115 (48.7%) patients were required chest tube insertion, and 95 (40.3%) patients needed blood transfusion. The pattern of chest injuries were predominated by ribs fractures (63.5%), lung contusion (59.3%) and pneumothorax (55.5%) and other injuries (Table-II). Single or multiple extra-thoracic injuries involving the head, abdominal organs, pelvis and limbs was seen in189 patients (80%).

**Table-II:** Pattern, Types of injury and Management approach of chest trauma cases seen in our institute, Riyadh, Saudi Arabia between 2013 -2018.

*Variables*	*N (%)*
***Trauma pattern***	
Blunt injury	225(95.3%)
Penetrating injury	11 (4.7%)
***Injuries Types of***	
Ribs fracture	150 (63.5%)
Left ribs fracture	102 (43.2%)
Right ribs fracture	88 (37.3%)
lung contusion	(59.3%) 140
Pneumothorax	131 (55.5%)
Left pneumothorax	83 (35.3%)
Right pneumothorax	83 (35.3%)
Hemothorax	80 (33.8%)
Left hemothorax	45 (19.1%)
Right hemothorax	43 (18.3%)
Flail chest	7 (3.0%)
Major vessels injury	17 (7.2%)
Pneumomediastinum	7 (3%)
Others injuries	21 (9.0%)
***Management approach***	
Chest tube	115 (48.7%)
Blood Transfusion	95 (40.3%)
Tracheostomy	36 (15.3%)
Emergency Thoracotomy	19 (8.0%)
Endo tracheal Intubation and Mechanical Ventilation	130 (55%)

**Fig.1:**
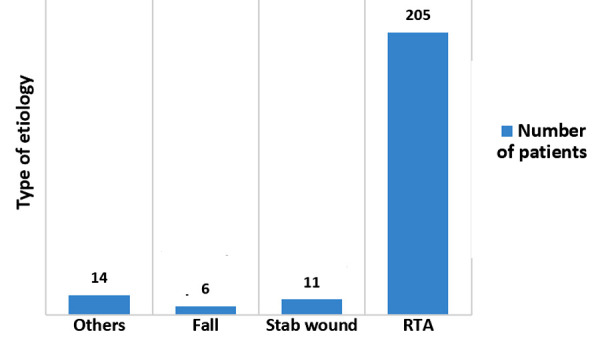
Etiology of trauma.

**Fig.2:**
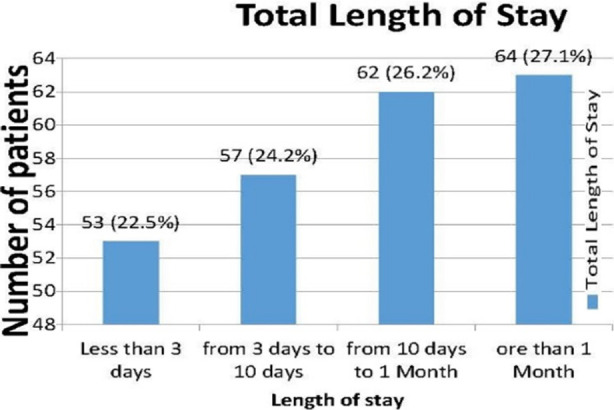
Total length of stay.

RTA was observed as the most common cause of chest injury (n=205, 86.9%) (Fig.1). Blunt trauma predominated the cases n=225 (95.3%). Hemothorax was the most common indication for emergency thoracotomy (n=15, 6.4%), which was caused by laceration of the lungs (n=11, 4.7%), as 19 patients (8%) of the total needed to have emergency thoracotomy (Table-III). Regarding the hospital stay, most of the patients stayed for more than three days in the hospital 183 (77.5%); (Fig.2).

**Table-III:** Indications and findings in thoracotomy.

*Variables*	*N (%)*
***Indications for thoracotomy***	
Hemothorax	15 (6.4%)
Chylothorax	2 (1.0%)
Bronchial injury	2 (1.0%)
***Findings in thoracotomy***	
Laceration of the lungs	11 (4.7%)
Tracheal injury	2 (1.0%)
Diaphragmatic injury	2 (1.0%)
Others(Massive surgical emphysema, Empyema, Thoracic duct injury, Cardiac injury)	4 (1.7%)

Finally, we have noticed that the worse outcome and significantly correlated with prolonged duration of hospital stay (P =0.010), endo tracheal Intubation and mechanical ventilation (P<0.001), Extra thoracic injury (p=0.032), Blood transfusion (P<0.001), Chest tube insertion (P<0.001), Lung contusion (P=0.040), tracheostomy (P=0.002), parenchymal lung injury (P=0.007), flail chest (P=0.029).

A linear regression analysis indicated that patients with endo tracheal Intubation and mechanical ventilation (B=0.240, beta=0.210, t=3.014, P=0.003), chest tube insertion (B=0.169, beta=0.149, t=2.399, P=0.017), parenchymal lung injury (B=0.219, beta=0.080, t=2.350, P=0.020), and lung contusion (B=0.196, beta=0.132, t=2.121, P=0.035) had significantly the worse outcomes. (Table-IV).

**Table-IV:** Pearson correlation and linear regression of variables related to worse outcome.

*Pearson correlation*			

*Variables*	*Pearson correlation coefficient*		*p value*
Longer duration of hospital stays	0.168		*0.010
Endo tracheal Intubation and Mechanical Ventilation	0.353		*<0.001
Extra thoracic injury	0.140		*0.032
Transfusion of blood	0.288		<0.001
Chest tube insertion	0.244		<0.001
Lung contusion	0.134		*0.040
Tracheostomy	0.206		*0.002
Parenchymal lung injury	0.176		*0.007
Flail chest	0.143		* 0.029
Linear regression			

*Variables*	*beta*	*t*	*p value*

Endo tracheal Intubation and mechanical ventilation	0.210	3.014	*0.003
Chest tube insertion	0.149	2.399	*0.017
Parenchymal lung injury	0.080	2.350	*0.020
Lung contusion	0.132	2.121	*0.035

## DISCUSSION

Chest trauma is a serious public health issue worldwide with RTA accounting for majority of these cases. In KSA, RTA accounts as one of the highest incidence of all international accidents.^2^-^5^ KSA has documented 86,000 mortalities and 611,000 injuries in the last two decades. A systematic review of the existing evidence of road safety and (RTA) in Saudi Arabia showed disparity and difference in the type of reporting of (RTA), outcome measures, and possible causes over a period of 25 years. All research exclusively looked into the drivers’ faults rather than the preventing measures.^6^

In our study, we assess the patterns, etiology, management and outcome of chest trauma cases in one of the main tertiary university hospital in Riyadh, the capital of Saudi Arabia. Saudi Ministry of Health (MOH) hospitals stated that 20% of all its beds, are occupied by RTA victims, and RTA account for 81% of deaths in these hospitals.^6^ Most of the reported studies and the literatures suggested that RTA is the leading cause of chest trauma in most of the series which was like our study (n=205, 86.9%).^7^,^8^

In contrast, one study conducted in Madrid, Spain indicated that falls was the most frequent cause of thoracic injuries followed by RTA.^9^ All the studies reported that most common type of chest injuries are blunt chest trauma.^6^-^12^

In our study, young adults’ males with a mean age of 32 years old were found to be more prone to RTA, this could be related to the legal system and regulations in Saudi Arabia, as previously prohibits women from driving. Furthermore, this could also due to most of the Saudi population belong to the younger age group and may be this group is predisposed to reckless driving behavior. Few studies, like our findings, indicated that injured patients were more likely to be male with mean age of 42 years compared to the female group.^13^-^14^

The clinical features of the thoracic trauma differ and varies from a simple injury to life-threatening condition. Rib fractures, lung or pulmonary contusion, pneumothorax and Hemothorax are the most common findings.^15^-^16^ Mattox indicated in his study that patients who underwent emergency thoracotomy due to traumatic hemothorax and laceration of the lungs and consequent loss of more than one liter of blood, were consistent of (5%- 10%) of all the chest trauma patients.^17^ In the current study, 19 patients (8%) underwent emergency thoracotomy, most frequently due to massive Hemothorax and Lung laceration.

Many studies have reported that the need for emergency thoracotomy will increase the duration of hospital stay, and both factors indicate more severe injury, thus a worse outcome.^18^-^20^ However, some recent studies have demonstrated that video assisted thoracoscopic surgery approach in thoracic trauma patients is a safe and effective method of management, as it could reduce the post-operative pain of these patients, accelerate the recovery, and reduce the incidence of wound infection in the treatment of chest trauma patients.^21^

Endo tracheal Intubation and mechanical ventilation, chest tube insertion, presence of parenchymal lung injury and lung contusion significantly correlated with worse outcomes and prolonged hospital stay. Intubation and mechanical ventilation is necessary and indicated when there is severe pulmonary injury and hypoxia regardless of the number of rib fractures, flail chest, lung contusion or the age of the patient. In fact, not all patients with flail chest injuries required mechanical ventilation, unless there is an associated severe pulmonary injury and hypoxia or breathing restriction that warrants intubation and mechanical ventilation.^20^

The presence of extra thoracic injuries, major vessels injuries, or cardiac injuries were found to prolong the mechanical ventilation time, and to predict a poor outcome. However, requiring massive blood transfusion, also translates to a higher mortality, as suggested by Huber et al and others.^20^-^22^

In addition, in our study, we have found that the total length of stay in the hospital exceeded more than three days was as many as 77.5% of the patients, indicating the burden of chest trauma injury and its associated morbidity, like increase the incidence of complications like chest infection, in addition the accounting for a high admission costs.

Furthermore, the findings in our study are similar to other studies done by Pasha or Huber et al, ^7.22^ which they have found that the associated extra thoracic injuries, Mechanical ventilation requirement and increased hospital stay (ICU admission) significantly increases the morbidity and mortality.

Finally, the use of this data from a single institute could have limited factor of the generalization of our study findings, although a general and accurate overview of the current local scenario could be obtained. Therefore, additional studies with larger sample sizes, including more cases of trauma from many centers or hospitals across all regions of the Kingdom of Saudi Arabia are needed for better understanding of the patterns, etiology, and management approaches.

As most of the causes of the chest trauma are due to RTA, it is essential to adopt a governmental preventive measures and protocols to reduce the trauma in general and the chest trauma deaths in particular. Like avoiding over speeding and following speed limits, the use of seat belts and child restraints in cars, improving the roads’ visibility, appropriate headlights and road lightings, obeying traffic rules, and the appropriate authority to make sure that the driver on the road is fit to drive a roadworthy vehicle.

## CONCLUSIONS

Chest trauma is a major health problem particularly in young adults’ males, RTA is the leading cause of chest trauma. The most common type of injuries were blunt trauma and chest injuries could be managed conservatively. Thoracotomy was most frequently due to hemothorax and lung laceration. Additional multi centers studies with larger sample sizes are needed for better understanding of patterns, etiology, and management approaches of chest injuries.

### Authors’ Contributions:

**WMH, SAN:** Research conceptualization, manuscript writing and are responsible le for integrity of the study.

**OSA, AHA, NHA:** Literature review and data analysis and **SAM:** Manuscript writing and editing.

All authors have read and agreed to the published version of the manuscript.
